# A Statistical Study on the Development of Metronidazole-Chitosan-Alginate Nanocomposite Formulation Using the Full Factorial Design

**DOI:** 10.3390/polym12040772

**Published:** 2020-04-01

**Authors:** Hazem Abdul Kader Sabbagh, Samer Hasan Hussein-Al-Ali, Mohd Zobir Hussein, Zead Abudayeh, Rami Ayoub, Suha Mujahed Abudoleh

**Affiliations:** 1 Department of Basic Pharmaceutical Science, Faculty of Pharmacy, Isra University, Amman 11622, Jordan; hazem.sabbagh@yahoo.com (H.A.K.S.); Zead.abudayeh@iu.edu.jo (Z.A.); rami.ayoub@iu.edu.jo (R.A.); abudoleh81@gmail.com (S.M.A.); 2 Department of Chemistry, Faculty of Science, Isra University, Amman 11622, Jordan; 3 Materials Synthesis and Characterization Laboratory, Institute of Advanced Technology (ITMA), Universiti Putra Malaysia, 43400UPM Serdang, Selangor, Malaysia

**Keywords:** full factorial design, optimization, metronidazole, nanocomposites, sodium alginate, chitosan

## Abstract

The goal of this study was to develop and statistically optimize the metronidazole (MET), chitosan (CS) and alginate (Alg) nanoparticles (NP) nanocomposites (MET-CS-AlgNPs) using a (2^1^ × 3^1^ × 2^1^) × 3 = 36 full factorial design (FFD) to investigate the effect of chitosan and alginate polymer concentrations and calcium chloride (CaCl_2_) concentration ondrug loading efficiency(LE), particle size and zeta potential. The concentration of CS, Alg and CaCl_2_ were taken as independent variables, while drug loading, particle size and zeta potential were taken as dependent variables. The study showed that the loading efficiency and particle size depend on the CS, Alg and CaCl_2_ concentrations, whereas zeta potential depends only on the Alg and CaCl_2_ concentrations. The MET-CS-AlgNPs nanocomposites were characterized by X-ray diffraction (XRD), Fourier-transform infrared spectroscopy (FTIR), thermal gravimetric analysis (TGA), scanning electron microscopy (SEM) and in vitro drug release studies. XRD datashowed that the crystalline properties of MET changed to an amorphous-like pattern when the nanocomposites were formed.The XRD pattern of MET-CS-AlgNPs showed reflections at 2θ = 14.2° and 22.1°, indicating that the formation of the nanocompositesprepared at the optimum conditions havea mean diameter of (165±20) nm, with a MET loading of (46.0 ± 2.1)% and a zeta potential of (−9.2 ± 0.5) mV.The FTIR data of MET-CS-AlgNPs showed some bands of MET, such as 3283, 1585 and 1413 cm^−1^, confirming the presence of the drug in the MET-CS-AlgNPs nanocomposites. The TGA for the optimized sample of MET-CS-AlgNPs showed a 70.2% weight loss compared to 55.3% for CS-AlgNPs, and the difference is due to the incorporation of MET in the CS-AlgNPs for the formation of MET-CS-AlgNPs nanocomposites. The release of MET from the nanocomposite showed sustained-release properties, indicating the presence of an interaction between MET and the polymer. The nanocomposite shows a smooth surface and spherical shape. The release profile of MET from its MET-CS-AlgNPs nanocomposites was found to be governed by the second kinetic model (*R*^2^ between 0.956–0.990) with more than 90% release during the first 50 h, which suggests that the release of the MET drug can be extended or prolonged via the nanocomposite formulation.

## 1. Introduction

Design of experiment (DE) is a systematic method in research used to determine the relationship between independent variables and response variables [1,2]. There are different types of DE, which include factorial designs [3,4,5], fractional factorial designs [6,7], full factorial designs (FFD) [8,9], Plackett–Burman designs [10,11], central composite designs (Box–Wilson designs) [12,13], Box–Behnken designs (BBD) [14,15], Taguchi designs (TD) [16,17] and response surface designs (RSD) [18,19].

In pharmacy, the term optimization can be defined as the process of discovering the best way of using the existing resources while taking into account all the parameters that influence the decisions of any experiment [20]. Modern pharmaceutical optimization involves a systematic design of experiments to improve drug formulation. The process begins with predicting and evaluating the independent variables that affect the formulation response and selecting the best response values. With optimization, the formulation steps and preparation that fulfill the desired characteristics of the final product could be minimized. 

Polynomial is one form of regression analysis. It is a non-linear analysis that correlates between the independent variable (*x*) and the dependent variable (*y*) as an *n*th degree polynomial in *x*. Different ways can be used in the fitting of the regression analysis for establishing approximate mathematical models. One of these fitting methods is called the stepwise method [21,22].It involves choosing the predictive variables by an automatic procedure [23,24]. In each step, a variable is added to or subtracted from the set of explanatory variables based on some pre-specified criteria.

After decades of basic nanosciences research, nanotechnology applications offer a wide range of opportunities in the fields of agriculture [24,25], food [26], environment [27,28] and drug delivery [29,30,31]. Nano-formulation technology has produced many new innovative drug delivery systems. Smart drug delivery, as well as polymeric nano-formulation as solid colloidal particles with diameters ranging from 1 to 1000 nm, preserves drugs against chemical decomposition and modifies drug release profiles in a controlled manner [25,26]. Polymeric nanoparticles are one example of nano-formulation. Research on polymeric nanoparticles has been especially focused on their role in drug delivery and drug targeting owing to their particle size and long circulation in the blood [27,28].They can be used therapeutically in vaccines, or as drug carriers, in which the drug can be encapsulated, entrapped, chemically attached, adsorbed or dissolved [29].

Chitosan and alginate, which are polymeric materials, were widely used in the development of nano-formulation products [30]. Both are non-toxic, stable hydrophilic polymers [31,32]. Chitosan-alginate has been used as a sustained release polymer matrix in different dosage forms [30,31,33,34]. Drug side effects may occur when administered in large quantities and sustained release formulations in nanocomposites by a single dose might be a suitable way to decrease drug complications due to its high concentration and increased patient compliance [35,36,37,38].

The drug used in this research, metronidazole (MET), is an antibiotic drug usually used to treat bacterial infections of the vagina, stomach or intestines, liver, skin, joints, brain, heart and respiratory tract. However, it is ineffective for viral infections (such as the common cold and flu). 

There have been many attempts by researchers to load MET using nano-formulations such as nanostructure lipid carriers (NLCs) [39], nano-emulsions [40], MET loaded into niosomes and then coated on dental implants using a layer-by-layer dip-coating technique with poly(lactic acid) (PLA) [41] and magnetic nanocomposites [42]. 

In the present study, the incorporation of MET into polymer nanoparticles was achieved. To the best of our knowledge, this work is reported for the first time, where MET as a guest drug was encapsulated into CS for the formation of CS-Algnanoparticleswith optimized preparation parameters.Traditionally, optimization is done by evaluating each factor independently. However, in a single-factor experiment, the interactions between important parameters are ignored. Response surface methodology (RSM) can be used to analyze the interactions between the different variables. The experimental data is input as a quadratic equation and the response is predicted. Stepwise regression analysis is one of the methodsthat can derive the best equation that can describe the data via surface or contour plots. Thus, in this work, Minitab softwareversion 18.1 and full factorial design were used to examine the effect of three independent variables (concentration of CS, Alg, and CaCl_2_) on three dependent variables (loading efficiency, particle size and zeta potential) for the synthesis of CS-Alg nanoparticles.

## 2. Materials and Methods

### 2.1. Materials

The chemicals used in this study are metronidazole (C_6_H_9_N_3_O_3_ (99% purity), Sigma-Aldrich, Taufkirchen, Germany), low molecular weight chitosan (10–120 kDa, 90% deacetylation, Sigma-Aldrich), and low viscosity sodium alginate (10–100 kDa, AZ Chem., Sigma Aldrich). All HPLCsolutions were used from VWR (West Chester, PA, USA). All other chemicals, including acetic acid and calcium chloride, were purchased from Chem CO (Port Louis, Mauritius).

### 2.2. Preparation of CS-Alg Nanoparticles and MET-CS-AlgNPs Nanocomposites

The method used was a modification of what is called the ionotropic pregelation method [43,44]. Solutions of CS, Alg and CaCl_2_ were first prepared. The pH of the CS and Alg solutions was adjusted to 5.5 and 5.0, respectively. The first step was the formation of the AlgNPs pre-gel, which was achieved by adding 6 mL of different concentrations of aqueous CaCl_2_ solution to 10 mL of Alg, followed by 30 min of stirring. The second step was the addition of 4 mL of CS solution to the AlgNPs pre-gel with stirring for another 30 min. The resultant solution was stirred overnight at room temperature to form uniform nanoparticles. The same procedure was used to form MET-CS-AlgNPs nanocomposites using only 100 milligrams of MET mixed with the Alg solution.

### 2.3. Methodology

First, the modeling of the responses (loading efficiency, particle size and zeta potential) was presented. Secondly, the FFD was built to perform the experiments. This was followed by the use of multiple regressions to develop the loading efficiency, particle size and zeta potential model responses. Finally, the analysis of concentration variance was used to analyze the experimental data to predict the effects and contribution of parameters on responses.

#### 2.3.1. Modeling of Different Responses

The loading efficiency percentis the first response, which was taken as a parameter and was defined as the amount of total entrapped drug divided by the total weight of the nanoparticles. The second and third responses measured were particle size and zeta potential. Table 1 shows the three independent parameters and their levels.

#### 2.3.2. Full Factorial Design

Full factorial design (FFD) is a method used by researchersto design experiments that consist of several factors with separate possible levels. With FFD, the experiment takes all possible combinations of the levels across all such factors. FFD allows researchers to study the effect of each factor, as well as their interactions, on the response variable [45]. In this study, the FFDwas used to conduct the experiments. Therefore, (2^1^ × 3^1^ × 2^1^) × 3 = 36 combinations were used, corresponding to *n* = 3 parameters or factors (CS, Alg and CaCl_2_) (Table 2).

### 2.4. MET Loading Efficiency

High-speed centrifugation was used to determine the loading efficiency (LE) of MET in the prepared nanocomposites, in which 2.0 mL of suspension were centrifuged (Hettich Universal 30 RF) at 10,000 rpm for 10 min, and the drug loading was measured by high-performance liquid chromatography (HPLC, Shimadzu, Japan), using a Venusil C18 column (4.6 mm × 250 mm, 5 μm) at 25°C. The UV detection wavelength was 323 nm, and the mobile phase was prepared by mixing acetonitrile/0.1% with phosphoric acid (5:95, *v*/*v*). The flow rate was 1.0 mL/min. The LE was calculated as follows:

(1) $$\%\;\text{Loading Effciency}\;(\mathrm{LE})=\frac{T_{p}-T_{f}}{\text{mass of nanoparticles}}\times 100$$

where T_p_ is the total mass of MET used to prepare the nanocomposites, and T_f_ is the free mass of MET in the supernatant.

### 2.5. Particle Size and Zeta Potential of Nanocomposites

Particle size and zeta potential of the nanocompositeswere analyzed through a dynamic light scattering (DLS) method using a Zetasizer Nano S (Malvern, UK) at the Arab Pharmaceutical Manufacturing. The analysis was performed in triplicates at a temperature of 25 °C.

For the particle size analysis, the samples were dispersed in distilled water, the cells were filled and capped and checked for the absence of any bubbles. 

The samples were prepared for zeta potential analysis by dispersing themin the distilled water and measuring the zeta values at 25 °C. 

### 2.6. Controlled Release Study of the MET from the Nanocomposites

The in vitro release of MET from the nanocomposites wasdetermined in a solution at pH 1.2, using a Perkin Elmer UV–Vis spectrophotometer with λ_max_ of 323 nm. A suitable amount of each nanocomposite was added to 2 mL of the media. The cumulative amount of drug released into the solution was measured at preset time intervals at corresponding λ_max_.

The percentage release of MET into the release media was calculated according to the formula:

(2) $$\%\mathrm{Release}=\frac{\mathrm{Concentration}\;\mathrm{of}\;\mathrm{MET}\;\mathrm{at}\;\mathrm{time}\;t\;(\mathrm{ppm})}{\mathrm{Concentration}\;\mathrm{corresponding}\;\mathrm{to}\;100\%\;\mathrm{release}\;\mathrm{of}\;\mathrm{MET}\;(\mathrm{ppm})}\times 100$$

The concentration corresponding to 100% release was obtained by adding a known amount of the nanocomposites into 2 mL HCl followed by sonicating and heating the nanocomposites at 37 °C.

### 2.7. Instrumentation

Powder X-ray diffraction (XRD) patterns were used to determine the crystal structure of the samples in the range of 2–70 degrees on an XRD-6000 diffractometer (Shimadzu, Tokyo, Japan) using CuK_α_ radiation (λ=1.5406 Å) at 30 kV and 30 mA at Universiti Putra Malaysia. Fourier transform infrared spectroscopy (FTIR) spectra of the materials were recorded over the range of 400–4000 cm^−1^ on a Perkin Elmer (model Smart UAIR-two). The thermogravimetric analysis was carried out using a Metter-Toledo 851e instrument (Switzerland) with a heating rate of 10 °C min^−1^, in 150 μL alumina crucibles and in the range of 30–900 °C. The zeta potential was measured at 25 °C by the dynamic light scattering (DLS) method using a Malvern Zetasizer Nano ZS (Malvern Instruments, Malvern, UK). UV–Vis spectra were measured to determine the release profiles using a Shimadzu UV-1601 spectrophotometer. 

## 3. Results and Discussion

### 3.1. MultipleLinear Regression Analysis

Multiple regression analysis is a statistical method that is used to estimate the correlation between dependent and independent variables. The term correlation coefficient (*R²*) indicates how well the data fit the multiple regression models. It provides a measure of how well-observed outcomes are replicated by the model, as the proportion of total variation of outcomes explained by the model. An *R²* close to 1 indicates that the regression model perfectly fits the data; the higher the *R²*, the more the dependent variations are explained by input variables and therefore the better the model. 

However, the demerit with *R²* is that it will stay the same or increase with the addition of more variables, even if they do not have any relationship with the output variables. This can be solved by using the “adjusted R square”, which is sensitive for adding variables that do not improve the model.

The linear (CS, Alg, and CaCl_2_), linear-square (CS*CS, Alg*Alg, and CaCl_2_*CaCl_2_) and linear-interaction equations (CS*Alg, CS*CaCl_2_ and Alg*CaCl_2_) have been fitted using a Minitab software for LE, particle size and zeta potential variables. The data was analyzed using stepwise regression which is a way to build a model by adding or removing independent variables, usually via a series of F-tests or T-tests. The variables to be added or removed are chosen based on the test statistics of the estimated coefficients.

Table 3 shows the ANOVA values for LE, particle size and zeta potential given in the suggested models. The *P*-value is less than 0.05, showing the model which is significant at a 95% confidence level. These LE, particle size and zeta potential models show that lack-of-fit error value is insignificant (0.283, 0.821 and 0.432, respectively) indicating that the fitted model is accurate enough to predict the response. The mathematical models were developed to determine the optimal values of the MET-CS-AlgNPs formulation conditions leading to maximum values of LE, minimumvalues of particle size and a negative value (~20 mV) of zeta potential.

The equations can be given in terms of the coded values of the independent variables as shown in the following Table 4.

Table 4 shows the regression model for three dependent variables for LE, particle size and zeta potential. The LE model showed that R-square values were found to be 98.91%, 98.68% and 99.35%, respectively. Moreover, the Adj-R-square values were found to be 98.62%, 98.19% and 99.02%, respectively.

### 3.2. Evaluation of the Models

#### 3.2.1. Pareto Chart of Responses of Standardized Effects and Normal Plot of the Standardized Effects

**Figure 1:**
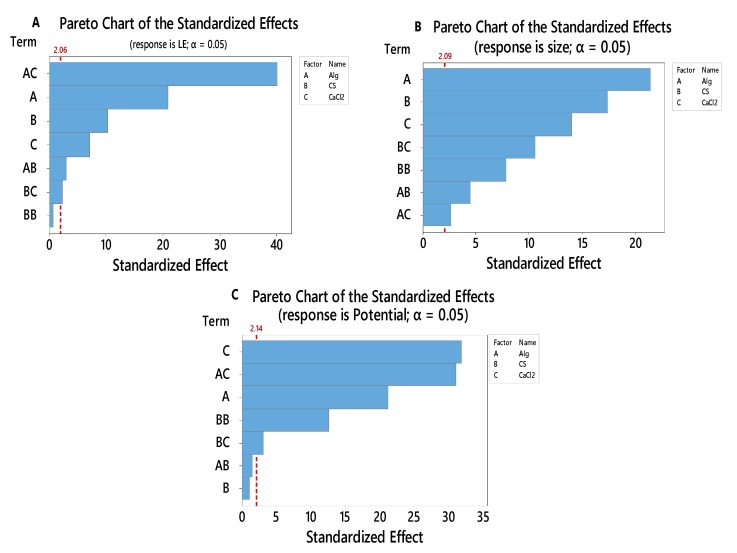
Pareto Chart of the standardized effects toward LE (**A**), particle size (**B**) and zeta potential (**C**).

A Pareto chart (Figure 1) is a graphical overview of the process factors and/orinteractions of influence, in ranking order from the most influencialto theleast influencial. A threshold line (*P*-value 0.05) indicates the minimum magnitude of statistically significant effects, considering the statistical significance of 95%.

Figure 1A indicates that the effect of BB i.e., CS × CS is statistically insignificant toward LE. The effect of AC (Alg × CaCl_2_) has the highest standardized effect on the LE followed by A, B, C, AB and BC. Hence, the term BB should not be considered for the empirical relation. The insignificance of BB can also be reasserted from the normal plot (Figure 1A), in which the points that do not fall near the fitted line are important. The factors having a negligible effect on the output response tend to be smaller and are centeredaround zero.

Figure 1B represents the effect of different parameters on particle size. The results indicate that all the effects are statistically significant. Factor A (Alg) has the highest standardized effect on the particle size followed by B, C, BC, BB, AB and AC. The significance of factors can be shown in the normal plot (Figure 1B).

Figure 1C shows the effect of different factors on the zeta potential response. The main factors (A, and C), square factors (B*B) and 2-way interaction (A*C and B*C) have a statistically significant effect on the response. C (CaCl_2_) has the highest standardized effect on the zeta potential followed by AC, A, BB, and BC. Hence, the terms AB and B should not be considered for the empirical relation.

#### 3.2.2. Contour Plot and Surface Plot of LE, Particle Size and Zeta Potential Against Selected Independent Variables

**Figure 2:**
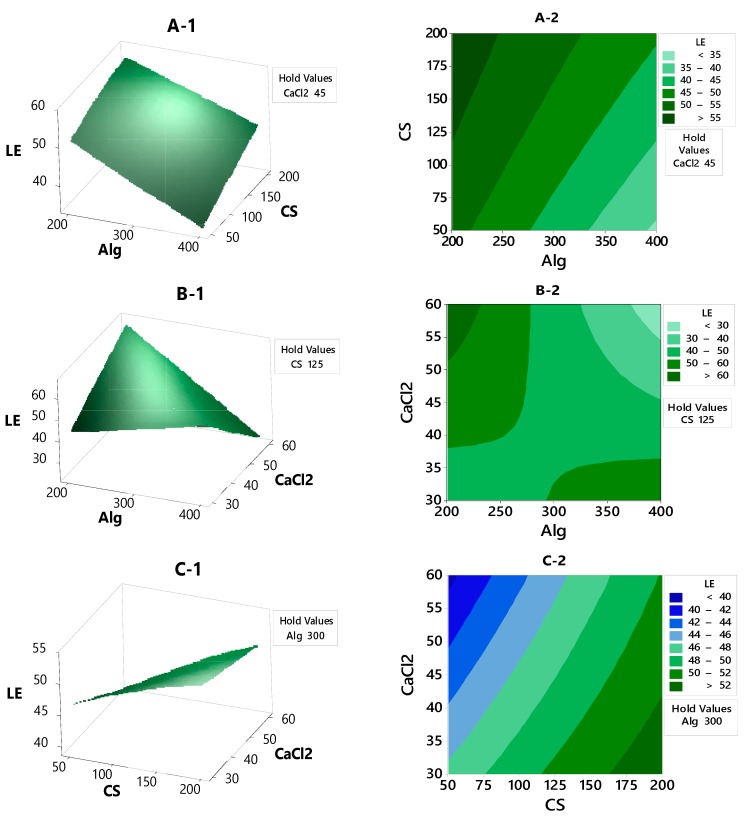
The contour plot and response surface of the LE with variances of CaCl_2_, Alg and CS concentrations.

The effect of the formulation and process variables on LE responsecan be evaluated by studying thecontour and response surface plots. Figure 2A-1,A-2 shows the response plots of LE as a function of CS and Alg concentrations, and it is seen to display a stationary ridge pattern. As the color gets darker, the LE response increases. The stationary ridge has a flat shape. Increasing the concentration of CS and decreasing the Alg can afford more space for LE (>55%). In Figure 2B-1,B-2, the contour and response surface plots show minimax patterns, with the stationary point (saddle point) being near the center of the design. From the stationary point (saddle point), increasing CaCl_2_ concentration while decreasing the Alg concentrationled to an increase in the LE response. Figure 2C-1,C-2 showsa flat shaped stationary ridge, and increasing the concentration of CS while decreasing CaCl_2_ concentration led to an increase of the LE by more than 52%.

**Figure 3:**
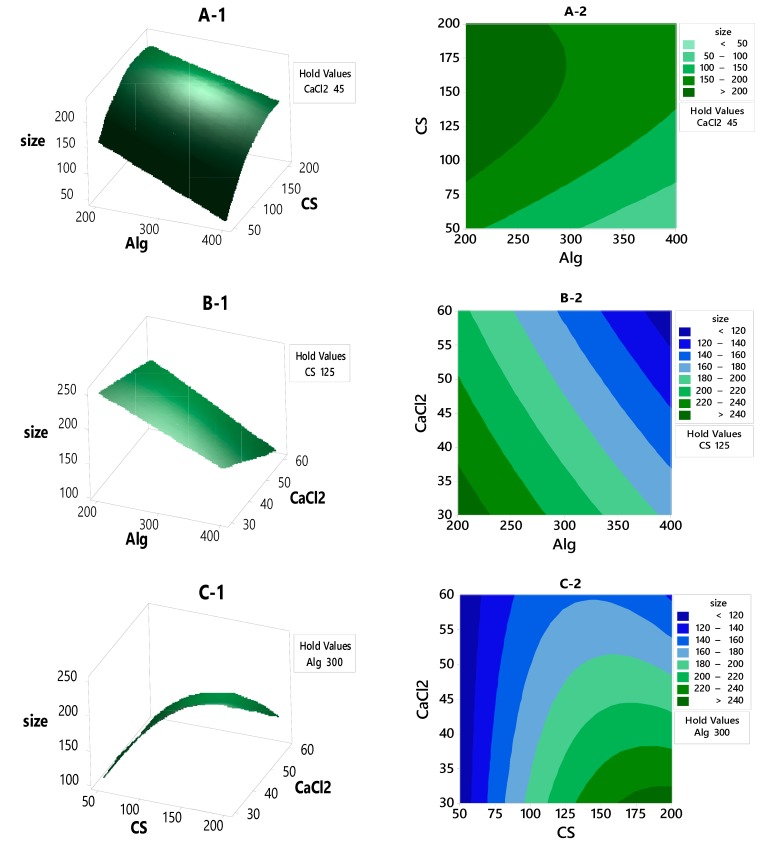
The contour plot and response surface of the particle size with variances of CaCl_2_, Alg and CS concentrations.

Figure 3A-1,A-2 shows a rising ridge pattern. As the color gets lighter, the particle size decreases. The minimum particle size was achieved using high concentrations of Alg and the lowest concentration of CS. From Figure 3A-2 it can be seen that the particle size below 50 nm can be prepared using 50 mg of CS and 400 mg of Alg. Figure 3B-1,B-2 shows that the particle size below 120 nm can be prepared by using 400 mg of Alg and 60 mg of CaCl_2_. In the case of CS and CaCl_2_ variables in Figure 3C-1,C-2, rising ridge pattern can also be seen. The particle size lower than 120 nm can be obtained using CS concentrations of 200 mg and CaCl_2_ concentrations ranging between 30 and 60 mg.

**Figure 4:**
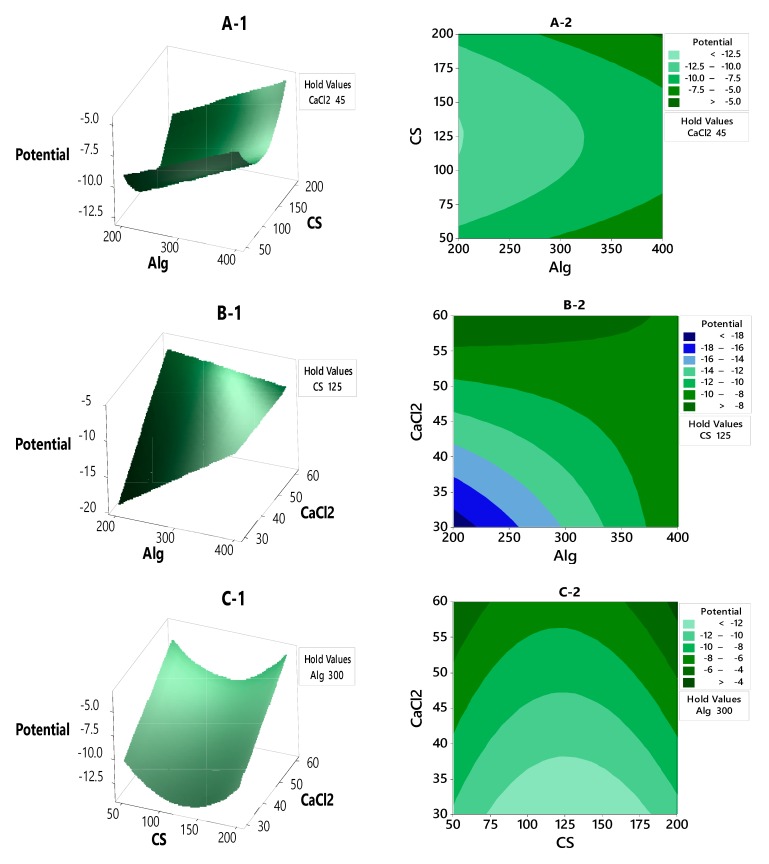
The contour plot and response surface of the zeta potential with variances of CaCl_2_, Alg and CS concentrations.

Figure 4 shows the3Dresponse surface and contourplots of the combined effect of CS, Alg and CaCl_2_on the zeta potential charge. The plots show that all the variables affect the zeta potential with rising ridge patterns. Figure 4A-1,A-2 shows the combined effect of Alg and CS concentrations; when the color gets lighter, the zeta potential becomesgreater than −12.5 mV, whereas when the color gets darker, the zeta potential becomesless than −5.0 mV. The zeta potential was higher than −5.0 mV when the Alg concentration was higherthan 300 mg and CS concentration was between 50–75 mg and 160–200 mg, whereas the zeta potential was lower than −12.5 mV when the concentration of Alg was less than 300 mg and the CS concentration was between 60–185 mg. 

Figure 4B-1,B-2 shows the contour plots of the effect of Alg and CaCl_2_ on the zeta potential. The zeta potential was between −8 and −18 mV; it was around −18 mV at low concentrations of both Alg and CaCl_2_, and around −8 mV at Alg concentrations between 200–350 mg with concentrations of CaCl_2_ between 55–60 mg.

Figure 4C-1,C-2 shows that the 3D surface and contour plots represent a rising ridge pattern. As the color gets darker, the zeta potential response reaches −4 mV; this occurs at high concentrations of CaCl_2_ of 55–60 mg and CS concentrations below 50 mg and higher than 200 mg. The zeta potential response at −4 mV can be achieved at a low concentration of CaCl_2_ of below 30 mg and a CS concentration between 75–175 mg.

#### 3.2.3. Main effects plot for LE, particle size and zeta potential

**Figure 5:**
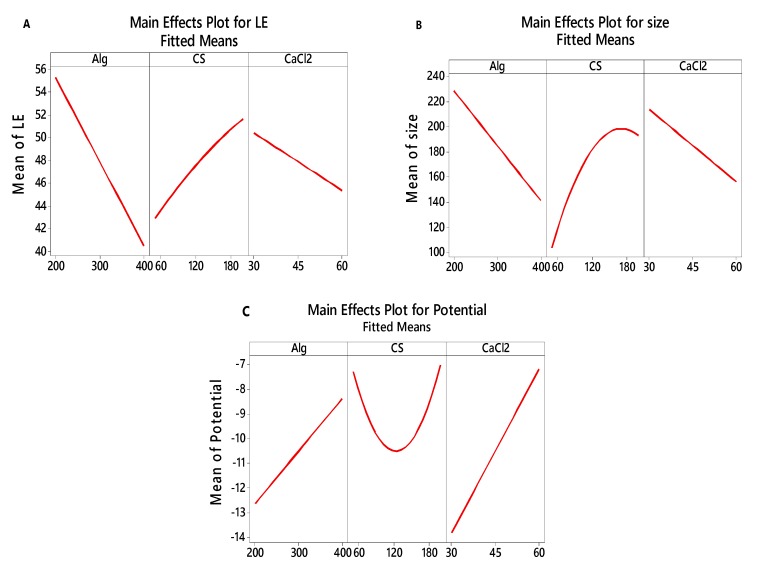
Main effects plot for LE, particle size and zeta potential.

Figure 5 shows a plot of the main effects (CS, Alg and CaCl_2_) used to examine differences between level means for LE, particle size and zeta potential factors. All factors seem to affect the LE, particle size and zeta potential because the line is not horizontal. Figure 5A shows that Alg at a concentration of 200 mg gave a higher LE (55%) compared to400 mg (40%). A CaCl_2_ concentration of 30 mg had a higher LE mean (50%) than the one at 60 mg (45%). The CS also affected the LE, with 200 mg of CS having had a higher LE mean (51%) than at 60 mg (43%). It is evident from Figure 5B that particle size is minimal (≈150 nm) at the highest level of Alg (400 mg)and CaCl_2_ (60 mg). In addition, the minimal particle size of approximately 100 nm can be obtained with the lowest level of CS (60 mg).

Based on the main effect plots in Figure 5C, the zeta potential was found to be the lowest at all of the highest values of Alg, CS and CaCl_2_ parameters tested. Both the parameters of Alg and CaCl_2_ concentrations show a linear potential pattern with an increase in their levels. However, CS concentration shows otherwise; although the highest level of CS concentration tested resulted in −7 mV potential, its mid-point shows a downward curvature in its response. The −11, −10 and −14 mV values of the mean zeta potential are observed at 200 mg of Alg, 120 mg of CS and 30 mg of CaCl_2_. From our studies, based on their potential data, the prepared nanocomposites were stable.

#### 3.2.4. The Interaction between the Factors thatAffects the LE, Particle Size and Zeta Potential

**Figure 6:**
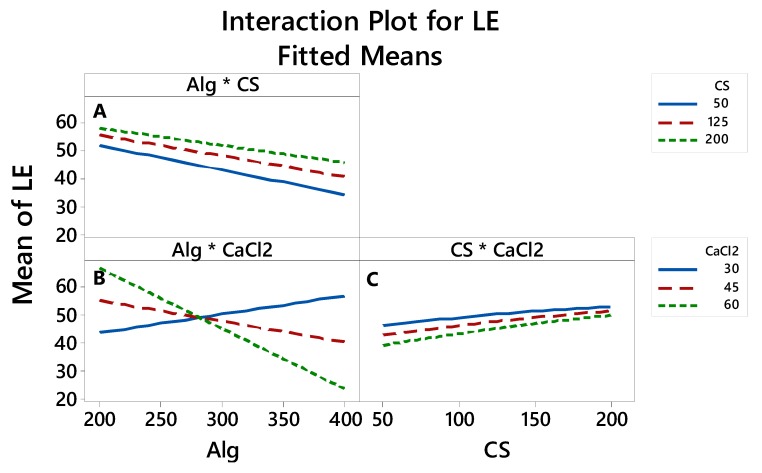
Interaction effects of factors on the loading efficiency.

**Figure 7:**
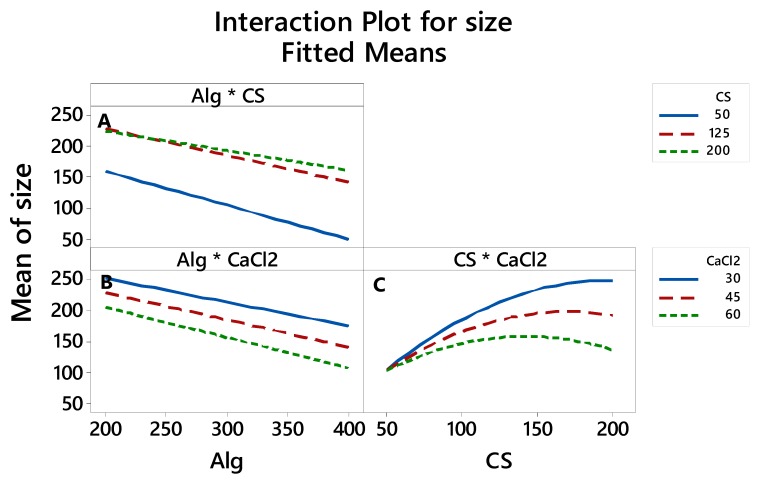
Interaction effects of factors on the particle size.

**Figure 8:**
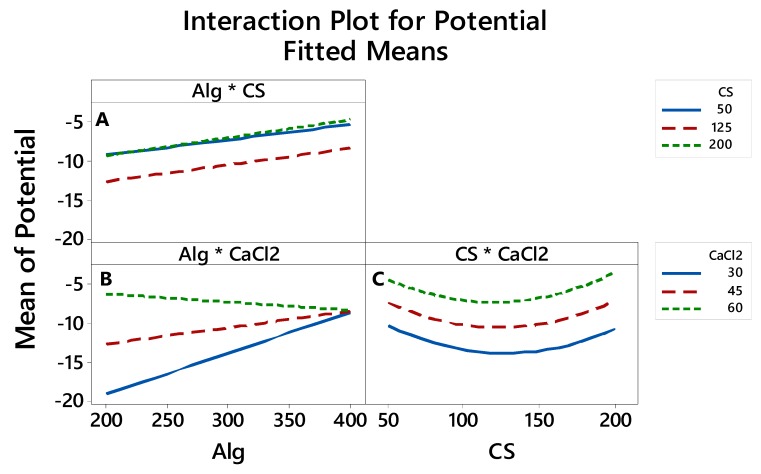
Interaction effects of factors on the zeta potential.

The interaction plots in Figure 6, Figure 7 and Figure 8 show how the relationship between one independent factor and a continuous response depends on the value of the second independent factor. The plot displays mean values for the levels of one factor on the x-axis and a separate line for each level of the other factor. The parallel lines in the interaction indicate that there is no relationship between the variables. When an interaction occurs, the lines are less parallel, and the strength of the interaction becomes greater.

In this interaction plot, the lines in Figure 6A are parallel, which indicates that there is a relationship between the variables. The interaction in Figure 6B has a nonparallel line, indicating that the relationship between Alg and LE depends on the value of CaCl_2_. For example, if 300 mg of Alg is used, then 30, 45 and 60 mg of CaCl_2_ are associated with the 50 % LE means, similar to that in Figure 6C.

Figure 7 shows that there is an interaction between the Alg*CS (Figure 7A) and CS*CaCl_2_ (Figure 7C). Figure 7A shows that there is a significant interaction between Alg and CS. The green and redlines (200 and 125 mg CS, respectively) show that the mean size response decreases when the Alg factor level is low, while in Figure 7C, the green, red and blue lines, which correspond to 60, 45 and 30 mg CaCl_2_, respectively, show that the particle size mean response decreases when the CS factor level is low. 

The interaction plotsshown in Figure 8A,B show the lines are not parallel, indicating that the relationship between Alg concentration and zeta potential depends on the value of CS (Figure 8A) and CaCl_2_ (Figure 8B). For example, when Algwas used at concentrations of 200 mg, then CaCl_2_ at 30 mg was associated with the −20 mV mean zeta potential (Figure 8B). However, when Alg with concentrationsof 200 mg was used, then CS at 50 and 125 mg was associated with −10 mV mean zeta potential (Figure 8A).

The Normal Plot of the Standardized effects, Normal probability plots, Residuals versus fitted value and Residuals versus observation toward LE, Particle Size and Zeta Potential (Figures S1–S4 in Supplementary Materials).

### 3.3. Optimization of LE, Particle Size and Zeta Potential

**Figure 9:**
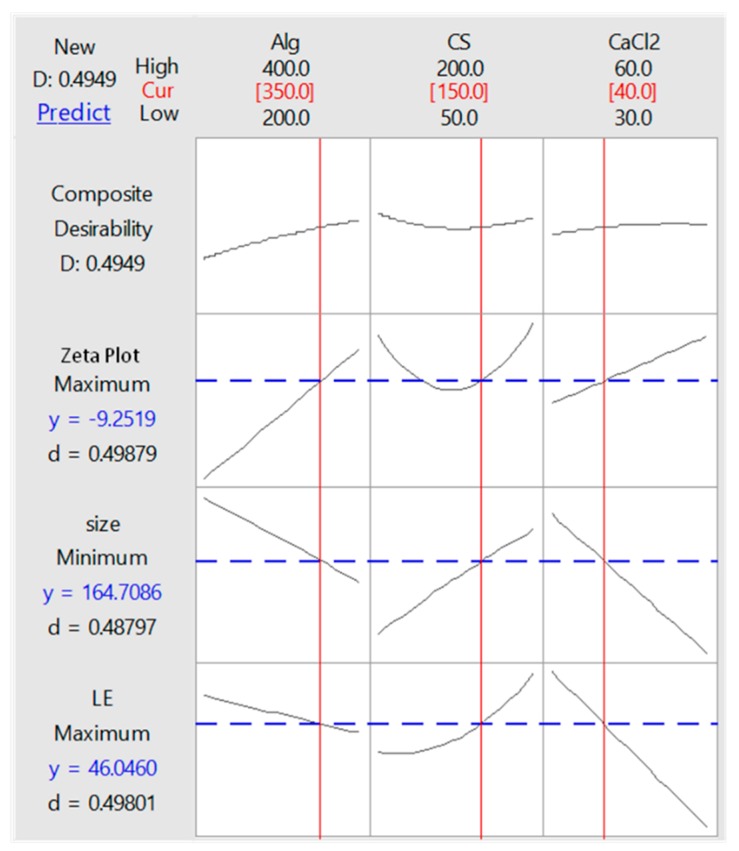
The optimization plot for metronidazole (MET), chitosan (CS) and alginate (Alg) nanoparticles (NP) (MET-CS-AlgNPs) nanocomposites.

In this study, the data was used to build a mathematical model such as linear, linear interaction, linear square and second-order model. Table 5 shows the selected mathematical model used to optimize the conditions of 46.05% for LE, minimizing the particle size to a 164 nm value and achieving a −9.25 mV zeta potential, using 350 mg Alg, 150 mg CS and 40 mg CaCl_2_ (Figure 9). 

### 3.4. Validation Test for Building Model

The comparison of experimental results with predicted values is shown in Table 6. From the table, the theoretical values for response were close to the experimentally obtained values. This result indicates that the mathematical models can be successfully used to predict the LE, particle size and zeta potential values for any combination of the Alg, CS and CaCl_2_ within the range of the performed experimentation.

### 3.5. X-Ray Diffraction of MET-CS-AlgNPs Nanocomposites

From the literature, the XRD diffractogram of CS shows crystalline properties with an intense peak at 2θ = 19.7°. At the same time, the XRD diffractogram of Alg shows semi-crystalline properties with a peak at 2θ = 13.6° [46].

**Figure 10:**
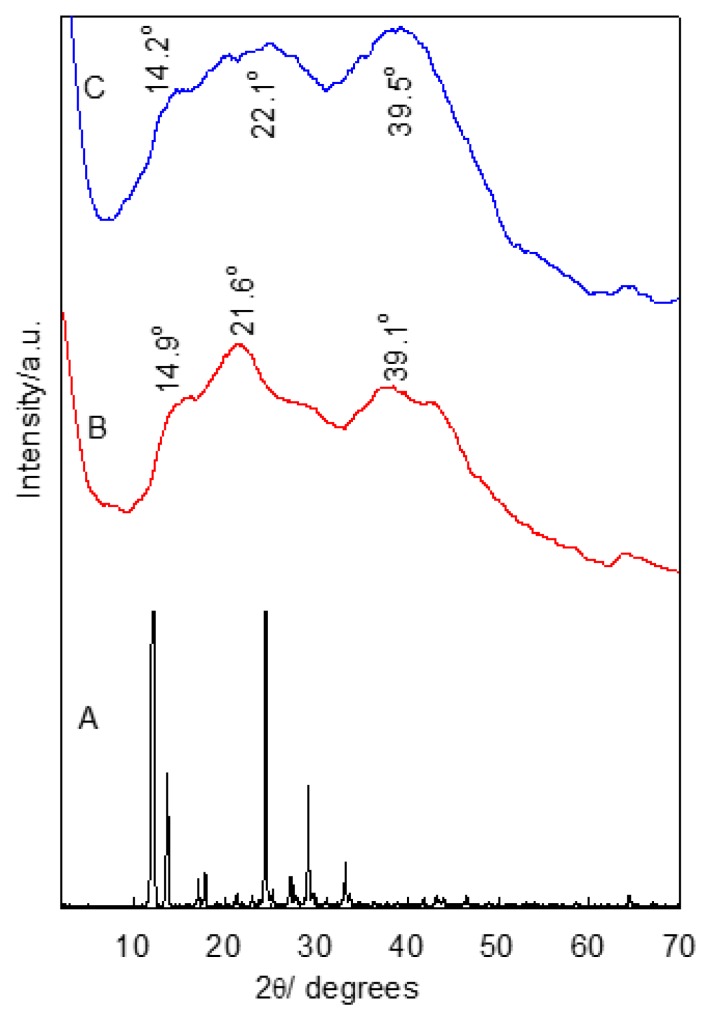
XRD diffraction spectra of MET (**A**), CS-AlgNPs (**B**) and MET-CS-AlgNPs (**C**).

XRD patterns of pure MET, CS-AlgNPs and MET-CS-AlgNPs nanocomposite formulations are illustrated in Figure 10A–C. The MET powder shows two sharp single peaks at 2θ = 11.0° and 22.3°,whereas the blank CS-AlgNPs nanoparticles gave a peak at 2θ = 14.9° and 21.6°, which indicates there is an amorphous pattern. The intensity of the diffraction peak of the CS-AlgNPs nanoparticles at 21.6° 2θ decreased after loading of MET and the peak for MET at 2θ = 11.0 and 22.3° disappeared in the MET-CS-AlgNPs nanocomposite. This might be due to the loading of MET inside the amorphous region of the nanocomposite matrix.

### 3.6. FTIR Spectroscopic Analysis of CS-AlgNPs and MET-CS-AlgNPs

**Figure 11:**
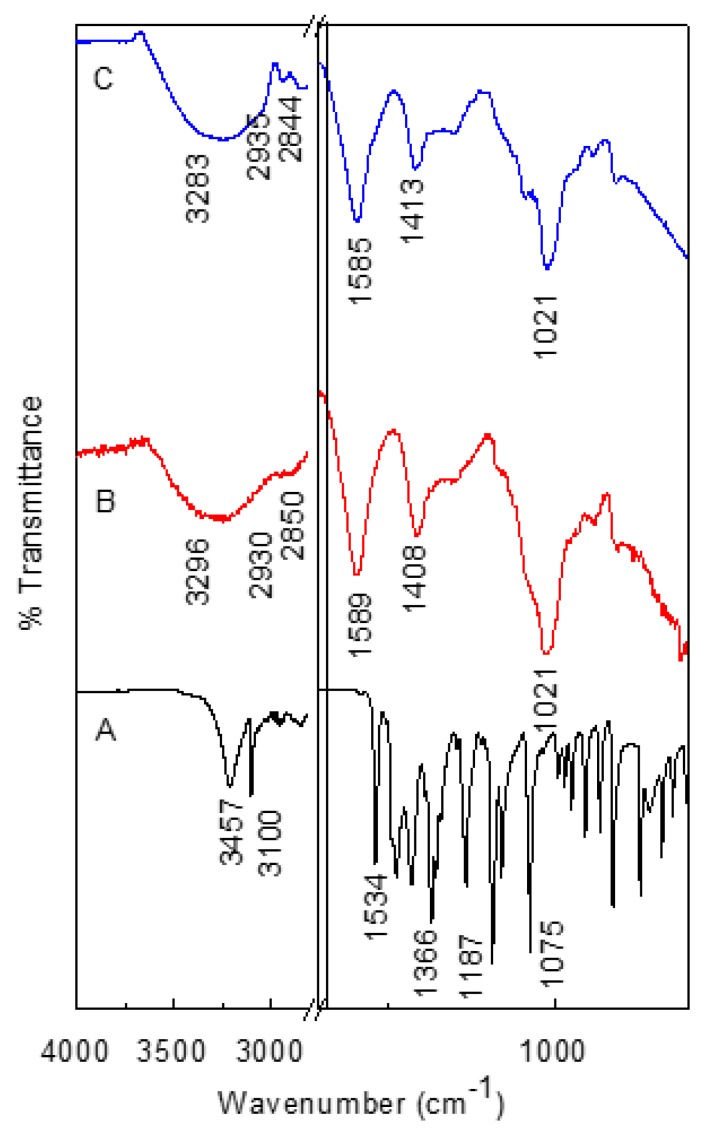
FTIR spectra of MET (**A**), CS-AlgNPs (**B**) and MET-CS-AlgNPs (**C**).

FTIR spectra of MET, CS-AlgNPs and MET-CS-AlgNPs are presented in Figure 11A–C. The FTIR spectra of pure MET (Figure 11A) show characteristic peaks at 3457 cm^−1^ (Hydroxyl –OH), 3100 cm^−1^ (C–C stretching), 1534 and 1366 cm^−1^)nitroso N–O stretch), and 1075, 875 cm^−1^ (C–N stretch) [47].

For CS-AlgNPs (Figure 11B), a band at 3296 cm^−1^ was observed due to O–H and N–H stretching. Absorptions due to vibration asymmetry CH_2_ and symmetry CH_2_ were located at 2930 and 2850 cm^−1^, respectively. A strong band near 1589 cm^−1^ corresponds to the C=O, C–N and N–H bending of amide I. Asymmetric stretching band of the COO^−^ group was centered near to 1420 cm^−1^ [48].

For MET-CS-AlgNPs (Figure 11C), some bands were downshifted; for example, from 3296to 3283 cm^−1^, from 1589 to 1585 cm^−1^ and from 1408 to 1413 cm^−1^. This can be explained due to the interaction between MET and CS-AlgNPs.

### 3.7. Thermogravimetric Analysis of MET-CS-AlgNPs Nanocomposites

**Figure 12:**
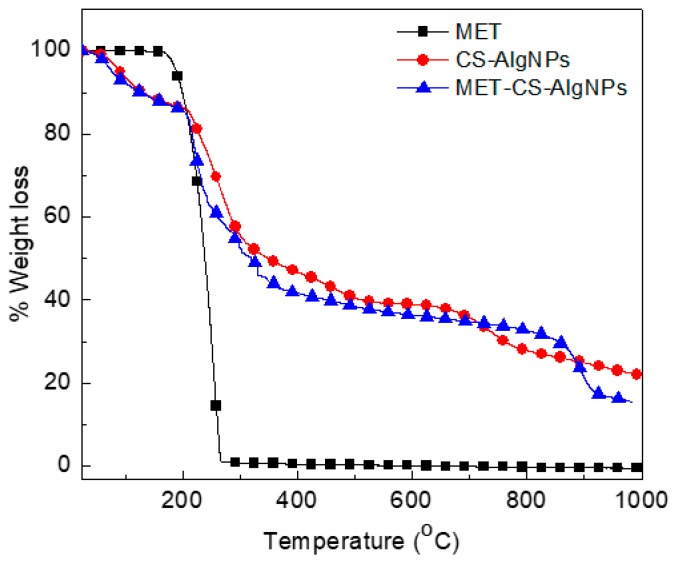
TGA curves of MET, CS-AlgNPs and MET-CS-AlgNPs nanocomposites.

The thermal decomposition process of MET-CS-AlgNPs nanocomposites and its pure counterpart CS-AlgNPs was evaluated by TGA/DTG analyses. These analysis curves give thepercentage weight loss due to the thermal decomposition (Figure 12). The results show that a pure MET sample undergoes a one-stage thermal degradation process, whileCS-AlgNPs and MET-CS-AlgNPs samples are degraded in a three-stage process. For the MET sample, the decomposition process occurred between 137–288 °C and with a mineral residue of 0.9% [49], which was due to the vaporization of volatile components [50].

The CS-AlgNPs show three main thermal stages; the first stage of the decomposition process occurred between 60–200 °C, which was due to the vaporization of volatile components, such as water molecules immobilized between chitosan chains during the coating process [51]. Based on the structure of CS and Alg, H_2_O molecules can be bounded by the hydroxyl group [52]. 

The second stage of weight loss, which occurred between 200–520 °C, is due to the release of water bound to the functional groups of CS and Alg polymers, which was not completely removed in the first step of the dehydration, and to the degradation of both polymers. 

A third inflection point occurred between 520–800 °C, which may be associated with the decomposition of functional groups of both polymers which were not completely removed by the previous stages. 

The TGA of MET-CS-AlgNPs (Figure 12) also shows three weight loss steps similar to CS-AlgNPs. The MET-CS-AlgNPs shows 70.2% weight loss compared to 55.3% for CS-AlgNPs. The extra weight loss is due to the incorporation of MET in the CS-AlgNPs.

### 3.8. Scanning Electron Microscopy

**Figure 13:**
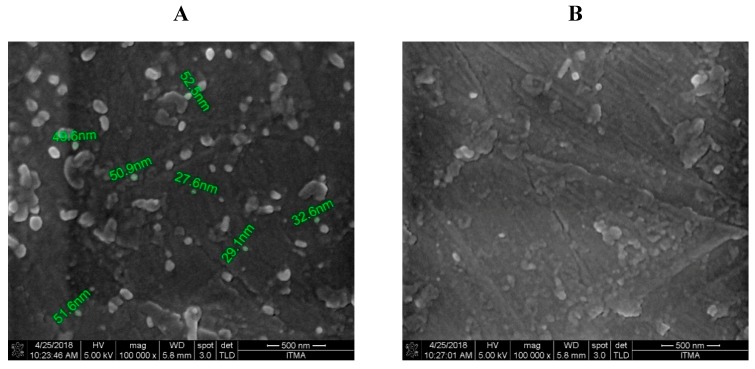
SEM micrographs of CS-AlgNPs (**A**) and MET-CS-AlgNPs (**B**) (100,000×).

The CS-AlgNPs and MET-CS-AlgNPs were morphologically characterized using the SEM (Figure 13). The micrographs of CS-AlgNPs (Figure 13A) show that the nanoparticles have a smooth surface with a spherical shape which is in agreement with previous studies [53]. Figure 13B shows that MET-CS-AlgNPsnanocomposites also have a spherical shape.

### 3.9. Transmission Electron Microscopy

**Figure 14:**
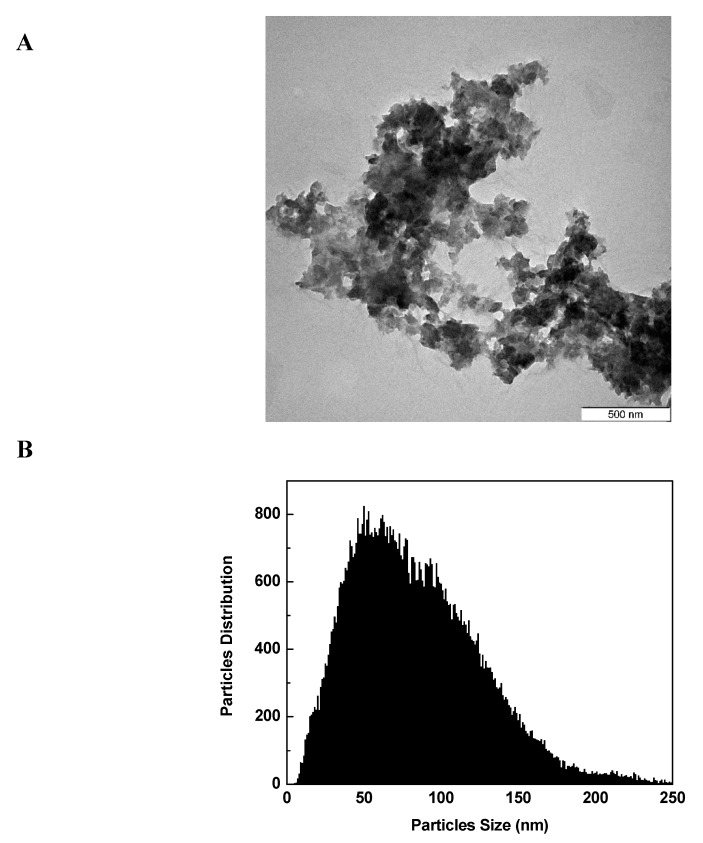
TEM images of MET-CS-AlgNPs (**A**) and their particle size distribution (**B**).

The MET-CS-AlgNPs nanocomposites were also examined using the transmission electron microscope (TEM), and the structure is as shown in Figure 14. From the Figure, it can be seen that the nanocomposites have irregular spherical shapes with agglomerate behaviors. The size of the main individual nanocomposites is around 80–110 nm.

### 3.10. Interactions between Chemical Components of MET-CS-AlgNPs Nanocomposites

**Figure 15:**
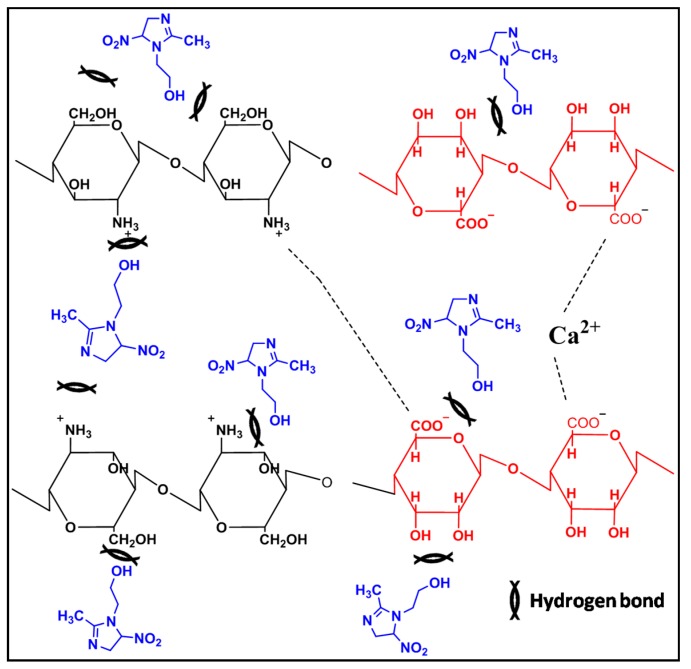
Possible interactions between components of MET-CS-AlgNPs nanocomposites.

Possible interaction between the components of the nanocomposites is shown in Figure 15. From the Figure, it can be seen that CS and Alg chains polymers are electrostatically held between positive charges of CS (protonated by acetic acid) and negative charges of Alg [54]. Moreover, calcium cations interact with negative charges of Alg. The structure of MET contains hydroxide (OH–) and nitro (NO_2_–) groups, which led to the formation of different hydrogen bonds with CS and Alg polymers (Figure 15).

### 3.11. Release Properties of MET from MET-CS-AlgNPs Nanocomposites

**Figure 16:**
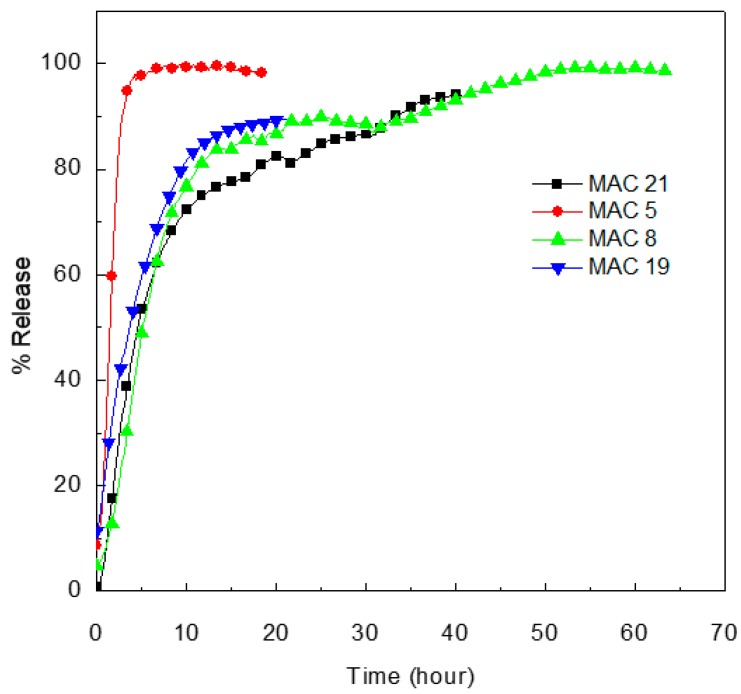
In vitrorelease behaviors of MET from MET-CS-AlgNPs nanocomposites in the 0.1 M HCl solutions.

The release profiles of MET-loaded CS-AlgNPs were obtained at 0.1M HCl (pH 1.2, to simulate physiological environments in the stomach). As shown in Figure 16, free MET was initially released very rapidly and almost 95% was released within 3.3 h for MAC 5 nanocomposite. This phenomenon is called the burst effect, and it may be due to the presence of the free drug in the nanocomposite. The MET release process from MAC 8, MAC 21 and MAC 19 nanocomposites was observed in two stages with sustained release properties. After 23 h, 90% of the MET was released from the MAC 19, whereas, after 40 hours, 90%, of the MET was released from the MAC 8 and MAC 21. The MAC 8 nanocomposite reached 97% release after 63 h. The MET release at 0.1M HCl could be explained by the enhanced solubility of CS at lower pH (1.2),which in turn promoted the diffusion of the MET through the pores of the AlgNPs matrix into the media [55,56]. These results suggest that the MET-CS-AlgNPs nanocomposites can be used in oral or intravenous administration.

The release kinetics of MET from MAC 8, MAC 21, MAC 19, and MAC 5 nanocomposites in 0.1M HCl were evaluated by fitting the data to various kinetic models (Table 7). Based on the highest adjusted R^2^, the best fitted model for all MAC 8, MAC 21, MAC 19, and MAC 5 nanocomposites was the second kinetic model with R^2^ values of 0.988, 0.956, 0.990 and 0.977, respectively. 

## 4. Conclusions

For the multiple linear regression analysis, the mathematical models for LE, particle size and zeta potential were developed using the responsesurface methodology to formulate the input parameters, which were Alg, CS and CaCl_2_ concentrations. Selected mathematical models showed that the developed response surface methodology models werestatistically significant and suitable for all conditions to have higher *R²* and adjusted R² values. High correlation values were determined between the experimental data and predicted ones.The concentrations of Alg, CS and CaCl_2_ with values of 350, 150 and 40 mg, respectively, were determined as the optimum conditions, resulting in the maximum LE (46.04%), the minimum particle size (164 nm) and the optimum zeta potential (−9.25 mV). The verification experiment was carried out to check the validity of the developed mathematical model that predicted LE, particle size and zeta potential within the range of 10% error limit and the prepared nanocomposites were generally stable.

In vitro MET release study of selected formulations; MAC 8, MAC 21, MAC 19, and MAC 5 showed 97%, 90%, 90% and 99% release in 60, 40, 20 and 10h, respectively. These results indicate that the nanocomposites could be effective in sustaining the MET release for a prolonged period.

## Figures and Tables

**Table 1:** Independent parameters and their levels.

Parameter		Levels (mg)		
		Low	Medium	High
A	Alg	200	-	400
B	CS	50	100	200
C	CaCl_2_	30	-	60

**Table 2:** Composition of formulations.

Std Order	Run Order	Sample Code	Alg	CS	CaCl_2_
17	1	MAC1	200	200	30
24	2	MAC2	400	200	60
10	3	MAC3	400	100	60
2	4	MAC4	200	50	60
35	5	MAC5	400	200	30
20	6	MAC6	400	50	60
32	7	MAC7	400	50	60
6	8	MAC8	200	200	60
22	9	MAC9	400	100	60
29	10	MAC10	200	200	30
36	11	MAC11	400	200	60
14	12	MAC12	200	50	60
25	13	MAC13	200	50	30
5	14	MAC14	200	200	30
9	15	MAC15	400	100	30
1	16	MAC16	200	50	30
31	17	MAC17	400	50	30
26	18	MAC18	200	50	60
3	19	MAC19	200	100	30
7	20	MAC20	400	50	30
16	21	MAC21	200	100	60
11	22	MAC22	400	200	30
28	23	MAC23	200	100	60
27	24	MAC24	200	100	30
13	25	MAC25	200	50	30
23	26	MAC26	400	200	30
30	27	MAC27	200	200	60
15	28	MAC28	200	100	30
34	29	MAC29	400	100	60
18	30	MAC30	200	200	60
12	31	MAC31	400	200	60
19	32	MAC32	400	50	30
4	33	MAC33	200	100	60
8	34	MAC34	400	50	60
33	35	MAC35	400	100	30
21	36	MAC36	400	100	30

**Table 3:** ANOVA values for loading efficiency (LE), particle size and zeta potential.

**LE model**								
	**DF**	**Adj SS**	**Adj MS**	**F value**	**Coef**	**T Value**	**VIF**	***P* value**
Model	7	9585.39	1369.34	337.95	47.908	67.86	-	0.000
Alg	1	1771.00	1771.00	437.07	−7.385	−20.91	1.04	0.000
CS	1	431.85	431.85	106.58	4.361	10.32	1.04	0.000
CaCl_2_	1	208.24	208.24	51.39	−2.532	−7.17	1.04	0.000
CS*CS	1	2.09	2.09	0.51	−0.605	−0.72	1.03	0.480
Alg*CS	1	236.52	136.52	9.01	1.252	3.00	1.04	0.006
Alg*CaCl_2_	1	6545.51	6545.51	1615.40	−13.998	−40.19	1.01	0.000
CS*CaCl_2_	1	22.71	22.71	5.60	0.987	2.37	1.04	0.026
Lack-of-fit	4	20.77	5.19	1.35	-	-	-	0.283
**Particle size model**								
Model	7	141548	20221.1	202.86	185.00	51.90	-	0.000
Alg	1	45889	45889.3	460.35	−43.50	−21.46	1.10	0.000
CS	1	30270	30270	303.67	44.42	17.43	1.12	0.000
CaCl_2_	1	19575	19574.9	196.37	−28.53	−14.01	1.12	0.000
CS*CS	1	6104	6103.7	61.23	−36.54	−7.83	1.13	0.000
Alg*CS	1	1963	1962.6	19.69	11.64	4.44	1.19	0.000
Alg*CaCl_2_	1	700	700.2	7.02	−5.43	−2.65	1.06	0.016
CS*CaCl_2_	1	11146	11145.6	111.81	−27.64	−10.57	1.17	0.000
Lack-of-fit	4	173	43.4	0.38	-	-	-	0.821
**Zeta potential model**								
Model	7	399.875	57.125	303.51	−10.501	−44.19	-	0.000
Alg	1	85.093	85.093	452.11	2.119	21.26	1.07	0.000
CS	1	0.256	0.256	1.36	0.133	1.17	1.19	0.263
CaCl_2_	1	191.991	191.991	1020.07	3.308	31.94	1.25	0.000
CS*CS	1	30.172	30.172	160.31	3.347	12.66	1.13	0.000
Alg*CS	1	0.404	0.404	2.15	0.164	1.47	1.17	0.165
Alg*CaCl_2_	1	181.922	181.922	966.57	-3.182	-31.09	1.22	0.000
CS*CaCl_2_	1	1.855	1.855	9.85	0.344	3.14	1.05	0.007
Lack-of-fit	3	0.562	0.187	0.99	-	-	-	0.432

DF: degrees of freedom, SS: sum of squares, F: F-test value and P: error variance.

**Table 4:** Regression model for dependent variables.

Regression Model	R-sq (%)	R-sq (adj)%
LE= −46.07 + 0.3252Alg − 0.0045 CS +2.5210CaCl_2_ 0.000108 CS*CS+0.000167Alg*CS − 0.009332Alg*CaCl_2_+ 0.000878 CS*CaCl_2_	98.91	98.62
Size= 96.7 − 0.4660Alg + 2.856CS + 2.256CaCl2 − 0.006495CS*CS + 0.001552Alg*CS − 0.00362 Alg*CaCl2 − 0.02457CS*CaCl2	98.68	98.19
Potential= −43.80 + 0.11391Alg − 0.1673CS + 0.8187CaCl2 + 0.000595CS*CS + 0.000022Alg*CS − 0.002121Alg*CaCl2 + 0.000305CS*CaCl2	99.35	99.02

**Table 5:** Response optimization plot for different responses.

Value	Alg (350 mg)	CS (150 mg)	CaCl_2_ (40 mg)
	Optimization Responses		
**LE**	46.0 ± 2.1%		
**Minimum Size**	164.71 ± 20.03 nm		
**Zeta potential**	−9.25 ± 0.51 mV		

**Table 6:** Response optimization for LE, particle size and zeta potential.

No.	Alg	CS	CaCl_2_	%LE			Particle Size (nm)			Zeta Potential (mV)		
				Exp	Theo	Error %	Exp	Theo	Error %	Exp	Theo	Error %
**1**	300	100	50	45.0	43.0	4.7	115	126	8.7	−9.5	−8.9	6.7
**2**	200	200	30	43.3	45.5	4.8	285	277	2.9	−14.5	−16.2	10.5
**3**	350	150	40	48.8	46.0	6.1	150	165	9.1	−10.8	−11.5	6.1

**Table 7:** The correlation coefficients (*R*^2^) obtained by fitting the MET release data from MET-CS-AlgNPs nanocomposites in aqueous solutions at 0.1M HCl [57,58,59].

Samples	R^2^			
	Pseudo-First Order	Pseudo-Second Order	Hixson-Crowell Model	Korsmeyer-Peppas Model
MAC 8	0.917	0.988	0.781	0.877
MAC 21	0.903	0.956	0.734	0.882
MAC 19	0.930	0.990	0.822	0.891
MAC 5	0.664	0.977	0.787	0.856
Equation	ln(*q*_e_ − *q*_t_) = ln*q*_e_ − *k*_1_*t*	*t*/*q*_t_ = 1/*k*_2_*q*_e2_ + *t*/*q*_e_	$\sqrt[{3}]{M_{o}}-\sqrt[{3}]{q_{t}}=\mathrm{Kt}$	$\frac{q_{t}}{q_{\infty }}={\mathrm{Kt}}^{n}$
	*q*_e_ is the quantity released at equilibrium, *q*_t_ is the quantity released at the time (*t*), *M*_o_ is the initial quantity of drug in the nanocomposite, *q*_∞_ is the release at the infinite time and *k* is the rate constant of the release kinetics			

## Supplementary Materials

The following are available online at https://www.mdpi.com/2073-4360/12/4/772/s1, Figure S1: Normal Plot of the Standardized effects toward LE (A), particles size (B) and zeta potential (C); Figure S2: Normal probability plots for LE (A), particles size (B), and zeta potential (C); Figure S3: Residuals versus fitted value for LE (A), particles size (B), and zeta potential (C); Figure S4: Residuals versus observation order for LE (A), particles size (B) and zeta potential (C).
