# Supplementary material for: A Statistical Study on the Development of Metronidazole-Chitosan-Alginate Nanocomposite Formulation Using the Full Factorial Design
Source: Polymers (Basel). 2020 Apr 1;12(4):772. doi: 10.3390/polym12040772 (PMC7240564; doi:10.3390/polym12040772)
Supplement: Supplementary file 1 [file polymers-12-00772-s001.pdf]

Raw data for ME-CS-AlgNPs

Alg	CS	CaCl2	Drug	LE		Alg	CS	CaCl2	Drug	size	CS	CaCl2	Drug		Alg	CS	CaCl2	Drug	Zeta Potential
200	50	30	100	41.5		200	50	30	100	142	50	30	100		200	50	30	100	-15.4
200	50	30	100	39.2		200	50	30	100	196	50	30	100		200	50	30	100	-15.2
200	50	30	100	42		200	50	30	100	167.7	50	30	100		200	50	30	100	-15.5
200	100	30	100	43.2		200	100	30	100	227	100	30	100		200	100	30	100	-10.4
200	100	30	100	42		200	100	30	100	224.4	100	30	100		200	100	30	100	-8.31
200	100	30	100	46		200	100	30	100	226	100	30	100		200	100	30	100	-6.13
200	200	30	100	47.1		200	200	30	100	266.1	200	30	100		200	200	30	100	-23.1
200	200	30	100	44		200	200	30	100	288.8	200	30	100		200	200	30	100	-16.2
200	200	30	100	43.5		200	200	30	100	276.1	200	30	100		200	200	30	100	-17.6
200	50	60	100	60		200	50	60	100	104.2	50	60	100		200	50	60	100	-6.83
200	50	60	100	61		200	50	60	100	110.2	50	60	100		200	50	60	100	-2.79
200	50	60	100	62		200	50	60	100	162.4	50	60	100		200	50	60	100	-0.9
200	100	60	100	65.1		200	100	60	100	195.4	100	60	100		200	100	60	100	-6.38
200	100	60	100	66		200	100	60	100	226.6	100	60	100		200	100	60	100	-18.6
200	100	60	100	68.5		200	100	60	100	211	100	60	100		200	100	60	100	-10
200	200	60	100	72.3		200	200	60	100	114.8	200	60	100		200	200	60	100	-2.41
200	200	60	100	70		200	200	60	100	169	200	60	100		200	200	60	100	-2.27
200	200	60	100	68		200	200	60	100	268.2	200	60	100		200	200	60	100	-7.79
400	50	30	100	50.3		400	50	30	100	63.2	50	30	100		400	50	30	100	-5.26
400	50	30	100	55		400	50	30	100	198.8	50	30	100		400	50	30	100	-4.86
400	50	30	100	51		400	50	30	100	131	50	30	100		400	50	30	100	-5.49
400	100	30	100	53.5		400	100	30	100	129.2	100	30	100		400	100	30	100	-7.54
400	100	30	100	55		400	100	30	100	152	100	30	100		400	100	30	100	-6.07
400	100	30	100	50.7		400	100	30	100	141.8	100	30	100		400	100	30	100	-8.27
400	200	30	100	63.7		400	200	30	100	233.2	200	30	100		400	200	30	100	-4.56
400	200	30	100	60		400	200	30	100	178.7	200	30	100		400	200	30	100	-4.87
400	200	30	100	66.2		400	200	30	100	211.9	200	30	100		400	200	30	100	-8.3
400	50	60	100	20.3		400	50	60	100	34.5	50	60	100		400	50	60	100	-6.22
400	50	60	100	18		400	50	60	100	32.2	50	60	100		400	50	60	100	-4.88
400	50	60	100	15		400	50	60	100	54	50	60	100		400	50	60	100	-3.54
400	100	60	100	21.9		400	100	60	100	86.3	100	60	100		400	100	60	100	-8.42
400	100	60	100	20		400	100	60	100	106	100	60	100		400	100	60	100	-7.74

400	100	60	100	20		400	100	60	100	96	100	60	100		400	100	60	100	-9.58
400	200	60	100	32.3		400	200	60	100	96.9	200	60	100		400	200	60	100	-4.3
400	200	60	100	30		400	200	60	100	95	200	60	100		400	200	60	100	-4.6
400	200	60	100	28		400	200	60	100	101.8	200	60	100		400	200	60	100	-4

1. Normal Plot of the Standardized effects

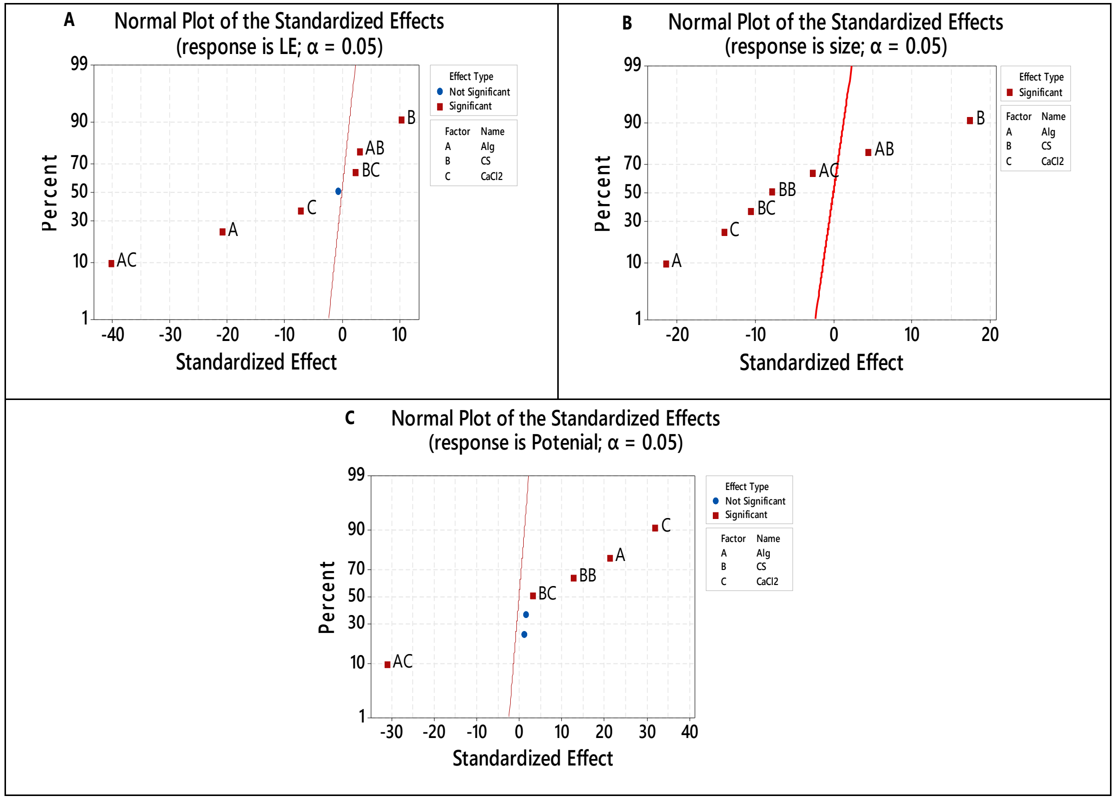

Figure S1. Normal Plot of the Standardized effects toward LE (A), particles size (B) and zeta potential (C).

2. Normal probability plot

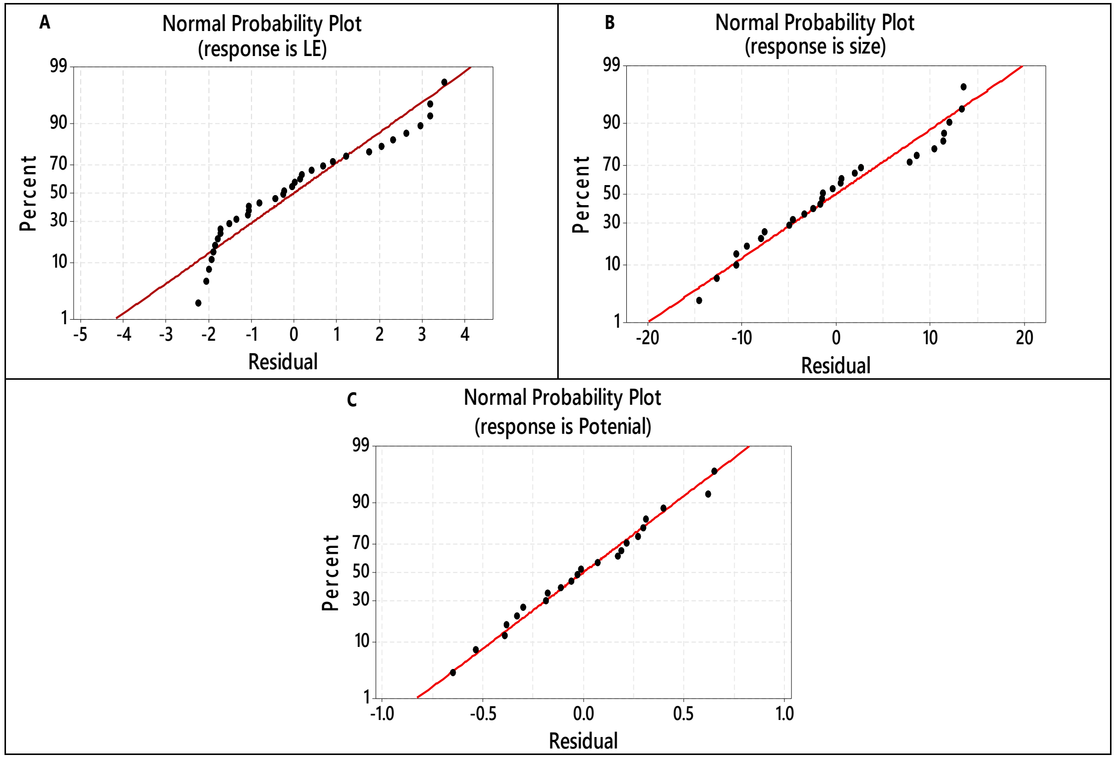

Figure S2. Normal probability plots for LE (A), particles size (B), and zeta potential (C).

3. Residuals versus fitted value

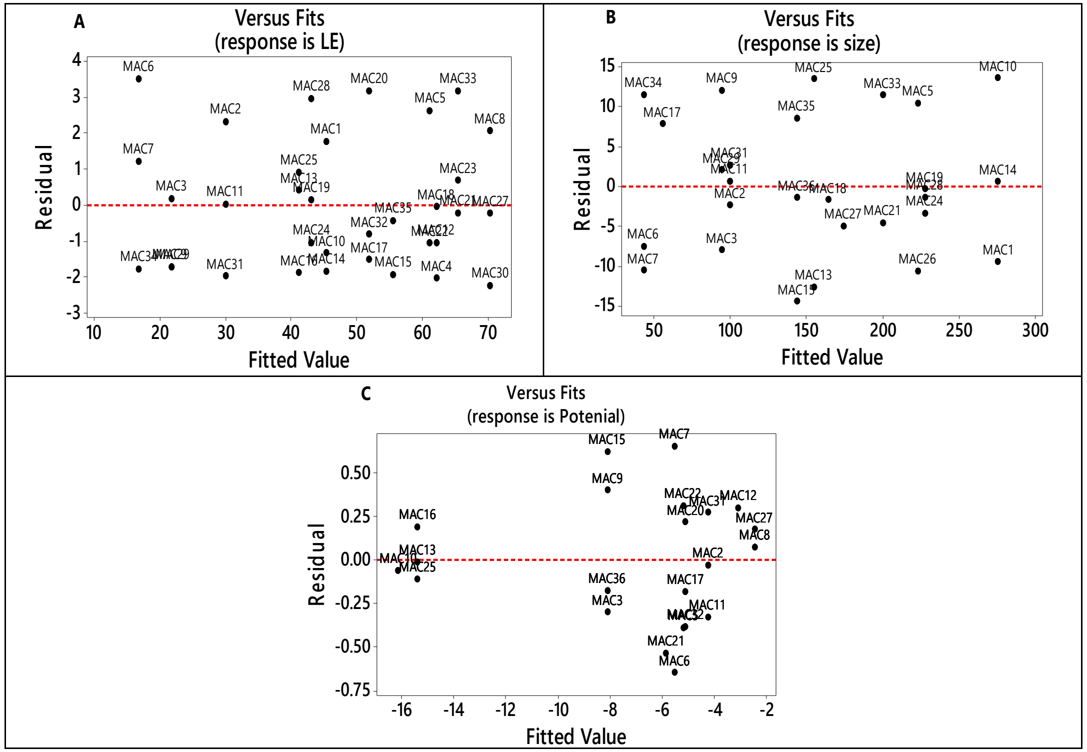

Figure S3 Residuals versus fitted value for LE (A), particles size (B), and zeta potential (C).

4. Residuals versus observation order

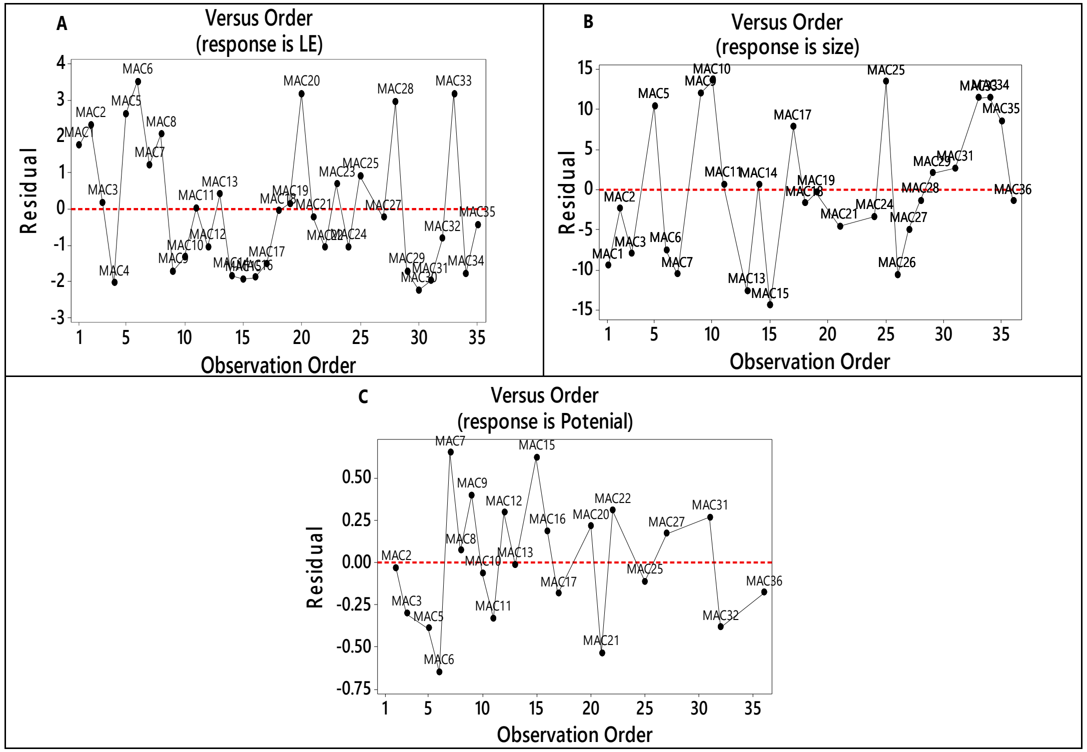

Figure S4. Residuals versus observation order for LE (A), particles size (B) and zeta potential (C).
